# Antibiotic-Dispensing Patterns and Awareness of Anti-microbial Resistance Among the Community Pharmacists in South-Central India

**DOI:** 10.7759/cureus.47043

**Published:** 2023-10-14

**Authors:** Moturu Dharanindra, Krishna Shriram Dhanasekaran, Supriya Rayana, Shaik Mohammad Noor, Piyush Bandela, Rudrapaka Pavan Sri Viswanadh, Kalva Hemanth Kumar

**Affiliations:** 1 Critical Care Medicine, Aster Ramesh Hospitals, Vijayawada, IND; 2 Research and Publications, Aster Ramesh Hospitals, Vijayawada, IND; 3 Clinical Pharmacy and Therapeutics, KL College of Pharmacy, Koneru Lakshmaiah Education Foundation, Guntur, IND; 4 Doctor of Pharmacy (PharmD) Program, KL College of Pharmacy, Koneru Lakshmaiah Education Foundation, Guntur, IND

**Keywords:** retail pharmacies, over the counter antibiotics, amr awareness, antibiotic dispensing pattern, antimicrobial resistance

## Abstract

Background

Anti-microbial resistance (AMR) is an ongoing epidemic contributing to extremely high healthcare costs and hospital admissions. Inappropriate dispensing of antibiotics is one of the root causes of AMR. Hence, our study aimed to assess antibiotic-dispensing patterns and AMR awareness among pharmacists from South-Central India.

Methodology

This cross-sectional observational study was conducted over a period of two months from June to July 2023. The pharmacies in urban and semi-urban areas of coastal and central districts of the Indian state of Andhra Pradesh were surveyed. Data were collected using a predesigned questionnaire for antibiotic-dispensing patterns and awareness of AMR, as approved by the Institutional Ethics Committee of Aster Ramesh Hospital, Vijayawada, India. The data were collected and analyzed descriptively by cross-tabulation.

Results

Among the 389 pharmacies that responded, 78% (*n* = 303) were dispensing antibiotics over the counter (OTC) and 22% (*n* = 86) were dispensing antibiotics only for valid prescriptions. It was found that antibiotics were dispensed OTC for common ailments such as the common cold, cough, sore throat, nasal congestion, fever, diarrhea, and urinary tract infections. As per the World Health Organization-recommended Access, Watch, and Reserve (AWaRe) criterion, antibiotics under the Watch group such as macrolides (azithromycin), fluoroquinolones (ciprofloxacin, norfloxacin, levofloxacin, and ofloxacin) and third-generation cephalosporins (cefixime and cefpodoxime) were found to be widely dispensed OTC. The most common antibiotics dispensed OTC were azithromycin (54.1%), amoxicillin (47.5%), cefixime (40%), amoxicillin + clavulanic acid (15.2%), ofloxacin (13.5%), ciprofloxacin (10%), and doxycycline (6.6%). Among the OTC dispensers, 82.5% (*n* = 250) were unaware of AMR and 17.5% were partially aware. However, 57% (*n* = 49) were unaware of AMR and its effects, in pharmacies dispensing antibiotics for valid prescriptions.

Conclusion

Our findings aggregate evidence on the alarming trend of inappropriate antibiotic-dispensing patterns that may further exacerbate AMR. Strict regulatory enforcement and periodical monitoring to regulate antibiotic dispensing to control unethical dispensing are inevitably necessary.

## Introduction

Antibiotics are one of the most commonly used drugs in the world and their improper use has become rampant [1]. The overuse and misuse of antibiotics are the contributing factors to the emerging anti-microbial resistance (AMR), which is now a global health emergency and likely an ongoing epidemic [2]. The mass production of antibiotics began a long time ago with the intention of preventing and treating infectious diseases. However, nowadays, many bacterial species have acquired the capacity to tolerate the effect of certain antibiotics by becoming resistant over time [3,4]. Prior studies denote that antibiotics could be obtained easily without prescriptions, that is, over the counter (OTC) widely across pharmacies in low- and middle-income countries (LMICs) [5,6].

India with a highly privatized healthcare system has a high incidence of drug-resistant pathogen-related deaths. It experiences an increased burden of infectious diseases annually. As a result of a twofold increase in antibiotic utilization, it has emerged as one of the most significant consumers of antibiotics globally. [7-9]. In India, only 0.7% of licensed doctors are present for every 1,000 inhabitants compared with 2.81 and 2.45 doctors in the United Kingdom and the United States, respectively [10]. As a consequence, Indian pharmacies are easily accessible and serve as the main source of healthcare. Whenever and wherever access to physicians is limited, patients directly reach the pharmacies, especially in rural areas. It has been estimated that over 750,000 retail pharmacies in the country supply antibiotics OTC without valid prescriptions [11]. Hence, this study was conducted to assess the dispensing patterns of antibiotics among the pharmacies and to assess the awareness of AMR among dispensers in South-Central India.

## Materials and methods

Study design

This cross-sectional observational study was conducted over a period of two months (June to July 2023) focusing on the antibiotic-dispensing patterns in the pharmacies and the awareness of dispensers on AMR. Data were collected using a predesigned questionnaire as approved by the Institutional Ethics Committee of Aster Ramesh Hospital, Vijayawada, India (approval no. DRCMSH/IEC/RR2019/2023/008).

Study site and population

The qualified pharmacists of the pharmacies in the urban and semi-urban areas of the coastal and central districts (such as NTR, Krishna, Eluru, Guntur, Vishakhapatnam, Kakinada, Palnadu, Ankapalli, Kurnool, East Godavari, and West Godavari) of the Indian state of Andhra Pradesh were surveyed in our study.

Study tools

Data were collected and recorded using the pharmacy interview/questionnaire method to assess the dispensing pattern of antibiotics. Five researchers from the Clinical Pharmacy field approached and collected data from the pharmacies with predesigned questionnaires that focused on the objectives of the study. The pharmacists were interviewed with open-ended questions such as the pattern of dispensing antibiotics (with or without prescription), antibiotic drugs dispensed OTC (without prescription), and the advice they gave to the patients on the course of antibiotic use. The dispensers were asked for their knowledge and awareness about AMR.

Study outcomes

The primary objective was to analyze the antibiotic-dispensing patterns among pharmacies and their dispensers. The secondary objectives were to assess the types of antibiotics dispensed OTC, advice on their course of intake, and awareness of the dispensers about AMR.

Sample size estimation

There are approximately 5,241 pharmacies in the Indian state of Andhra Pradesh. The sample size was calculated based on previous surveys in other Indian states [12-14]. A minimum sample size of at least 250 is needed for determining the prevalence of non-prescription-based anti-microbial drug sales with a confidence level of 95% and a margin of error of 5%, assuming a 50% prevalence rate [12-14]. By convenience sampling, we approached a total of 427 retail pharmacies of which 389 pharmacies with qualified pharmacists responded.

Statistical analysis

The responses were collected in predesigned Google Forms recorded in spreadsheets and double checked for errors. All the categorical variables expressed in numbers (*n*) and frequencies (%) were analyzed descriptively. The sample size was based on the maximum number of pharmacies (≥250) that could be approached over the study duration. Data were expressed and analyzed descriptively by cross-tabulation using IBM SPSS Statistics, Version 22.0 (IBM Corp., Armonk, NY).

## Results

Initially, a total of 427 retail pharmacies in the coastal and central districts of Andhra Pradesh were randomly selected and surveyed. Among them, 389 pharmacies that fully responded to the survey were included in the study.

Practice toward OTC sale of antibiotics

Among the 389 pharmacies included, 78% (*n* = 303) of pharmacies responded that they were dispensing the antibiotics OTC, that is, selling antibiotics without a valid prescription. It was found that only 22.1% (*n* = 86) followed the pattern of dispensing antibiotics with a valid prescription from a registered medical practitioner (Table 1).

**Table 1:** Antibiotic-dispensing patterns of pharmacies and awareness of AMR among their dispensers. AMR, anti-microbial resistance; OTC, over the counter.

S. No.	AMR Awareness	Overall Awareness (*n *= 389)	Antibiotic-Dispensing Pattern	
			OTC/without prescription (*n *= 303)	Only with prescription (*n *= 86)
1.	Aware, *n* (%)	90 (23)	53 (17.5)	37 (43)
2.	Unaware, *n* (%)	299 (77)	250 (82.5)	49 (57)

Common OTC-dispensed antibiotics

Most registered pharmacists/dispensers responded that they dispense antibiotics OTC for common ailments such as the common cold, cough, sore throat, nasal congestion, fever, diarrhea, and urinary tract infections (UTIs).

Azithromycin (54.1%) was identified as the most common antibiotic dispensed OTC. It was followed by amoxicillin (47.5%), cefixime (40%), amoxicillin + clavulanic acid (15.2%), ofloxacin (13.5%), ciprofloxacin (10%), and doxycycline (6.6%) that were dispensed without valid prescriptions. The community pharmacists dispensed these drugs (symptomatically for minor ailments) to the patients OTC (Table 2).

**Table 2:** Antibiotic drugs dispensed over the counter (OTC) by pharmacies without valid prescriptions.

S. No.	Name of the Antibiotic	Responses on OTC Dispensing (*n *= 303); *n* (%)
1.	Amoxicillin	144 (47.5)
2.	Amoxicillin + clavulanic acid	46 (15.2)
3.	Cefixime	121 (40)
4.	Cephalexin	4 (1.3)
5.	Cefpodoxime	10 (3.3)
6.	Azithromycin	164 (54.1)
7.	Roxithromycin	4 (1.3)
8.	Doxycycline	20 (6.6)
9.	Ciprofloxacin	30 (10)
10.	Ofloxacin	41 (13.5)
11.	Norfloxacin	5 (1.6)
12.	Levofloxacin	1 (0.3)
13.	Metronidazole	5 (1.6)
14.	Tinidazole	1 (0.3)
15.	Neomycin	1 (0.3)
16.	Linezolid	1 (0.3)
17.	Rifaximin	1 (0.3)
18.	Clindamycin	2 (0.6)

Among the OTC-dispensed antibiotics, azithromycin, amoxicillin, and cefixime were most commonly dispensed for upper respiratory tract infections, whereas fluoroquinolones such as ofloxacin and ciprofloxacin were given commonly for acute gastroenteritis and UTIs. Pharmacists advised the patients to consult the physician if the symptoms persisted or worsened beyond the advised course of intake.

Awareness of dispensers about AMR

The community pharmacists/dispensers in the pharmacies were also asked for their knowledge and awareness of AMR. The questions asked to determine awareness and unawareness were as follows: (1) What is AMR? (2) Are you aware of the consequences of AMR? (3) Are you aware of the current regulations for the sale of antibiotics? Among 303 pharmacies practicing OTC dispensing of antibiotics, 82.5% (*n* = 250) were unaware of emerging trends of AMR, whereas, 17.5% of the dispensers were partially aware of AMR. Even though 22.7% (*n* = 89) of pharmacies dispense antibiotics only with valid prescriptions, 57% (*n* = 49) of their dispensers were unaware of AMR and its effects (Table 1).

Antibiotic intake course patterns advised by pharmacists dispensing antibiotics OTC

**Figure 1:**
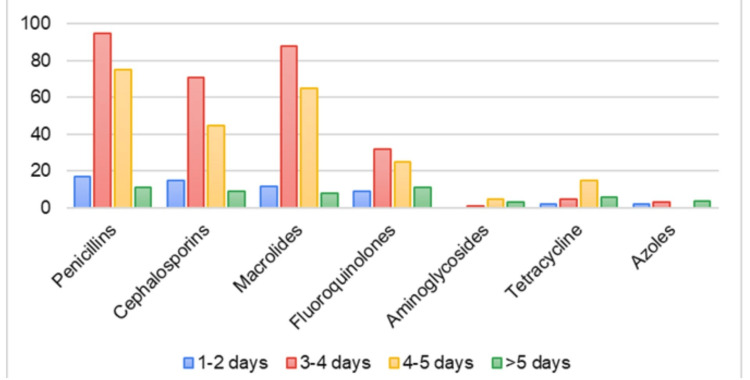
Antibiotics and their intake course patterns advised over the counter (OTC).

Most of the pharmacists dispensing antibiotics OTC were found to be advising inappropriate courses for antibiotic intake (i.e., number of days) (Figure 1). An intake course of one to two and three to four days was predominantly followed for the antibiotic classes such as penicillins, cephalosporins, macrolides, and fluoroquinolones. Only a few dispensers were found to be advising the patients with appropriate antibiotic regimens.

## Discussion

This community pharmacy-based survey was conducted in the central and coastal districts of Andhra Pradesh, India. Our study aimed to focus on the prevalence of OTC antibiotic dispensing and the awareness of dispensers about AMR. It was observed that OTC dispensing of antibiotics was more common among retail pharmacies and dispensers across Andhra Pradesh. In our study, approximately 78% of the pharmacists responded as they were dispensing antibiotics OTC without prescriptions. It was also observed that none of them asked the patients about their history of antibiotic use, drug allergies, or interactions.

Some of them dispensed antibiotics for minor ailments such as sore throat, nasal congestion, and common cold with/without the patient’s request. This finding was comparable with the findings of a study by Shet et al. in which 66.7% of pharmacies dispensed antibiotics without prescriptions [14]. In a study by Nafade et al., non-prescription antibiotic dispensing was low in the pharmacies of the Udupi district of Karnataka, India [15]. The differences in our results when compared with theirs might be due to the conduction of the study in a single district of a state. It is an open secret in India that most drugs can be obtained OTC from an easily approachable retailer. People find it easy to approach nearby pharmacy retailers for minor ailments, who are ready to oblige by handing over small quantities of various drugs, including a supply of antibiotics for two to three days, for immediate symptom relief [16].

It was observed that among the OTC dispensers, 82.5% were unaware of the emerging trends of AMR whereas 17.5% were partially aware of AMR. It was almost similar to the findings of a study by Tanveer et al., where 78% of dispensers in the Indian state of Telangana were unaware of AMR and its effects [17].

The World Health Organization has classified antibiotics into three categories to regulate their usage: Access, Watch and Reserve (AWaRe). The goal is to reduce the use of the Watch group and Reserve group antibiotics and to increase the use of Access group antibiotics [18]. Access group antibiotics show activity against a wide range of commonly encountered susceptible pathogens and have a lower potential of causing AMR than the antibiotics in the other two groups. The Access group includes antibiotics such as amoxicillin/ampicillin, benzathine penicillin, and trimethoprim/sulfamethoxazole. The Watch group includes antibiotics with a higher resistance-causing potential than the Access group and includes antibiotics such as third-generation cephalosporins, fluoroquinolones, and carbapenems. The Reserve group includes antibiotics that should be reserved only for the treatment of confirmed or suspected infections caused by multi-drug-resistant organisms [16,18].

In our study, the Access group antibiotics dispensed OTC were amoxicillin and amoxicillin + clavulanic acid. However, we found that the antibiotics under the Watch category were also widely dispensed OTC. These were fluoroquinolones (such as ciprofloxacin, ofloxacin, norfloxacin, and levofloxacin), third-generation cephalosporins (such as cefixime and cefpodoxime), and macrolides (azithromycin) (Table 2). This was similar to a study by Kotwani et al., conducted in the Indian states of Haryana and Telangana [16]. According to our findings, the most commonly dispensed antibiotics OTC were azithromycin (54.1%), amoxicillin (47.5%), and cefixime (40%). Our findings were almost similar to the study results of Shet et al. where amoxicillin was the most commonly dispensed antibiotic OTC followed by azithromycin and ciprofloxacin [14].

According to the Schedule H of Drugs and Cosmetic Rules of India, all antibiotic drugs have been regulated for sale only under a valid prescription from a registered medical practitioner [11,16,19]. In addition, Schedule H1 was introduced in 2013 to regulate the use of specific antibiotics (such as third- and fourth-generation cephalosporins, carbapenems, newer fluoroquinolones, and first- and second-line antitubercular drugs). Dispensing of both Schedule H and Schedule H1 drugs must be recorded in a separate register and maintained by pharmacies. The patient demographics with the prescribed drug’s name and quantity should be recorded for each dispense [19,20]. Knowledge is a key driver of professional practice, and pharmacists are the gateway to not only medicine use but to the larger healthcare system as well. When access to healthcare is limited, pharmacists ought to not only dispense medicines but also play a key role as stewards for appropriate antibiotic use in the community [21,22].

Even though the OTC dispensing of antibiotics is technically prohibited, these laws are yet to be implemented widely. Inappropriate antibiotic use, doses, and treatment duration all raise the possibility of certain microorganisms developing resistance [23,24]. Awareness about AMR (due to inappropriate use) and its future consequences are to be seriously taken [25]. The pharmacies and their dispensers should be monitored periodically and encouraged to practice ethical dispensing by national and state pharmacy regulatory authorities. Unethical drug dispensing practices (particularly for antibiotics and other Schedule H drugs) should not be tolerated.

Improper antibiotic dispensation is dangerous and results from a lack of specialized healthcare training, or informal dispensers [26]. The national pharmacy councils could take measures to upskill pharmacists by implementing continuing medical education programs on AMR. Pharmacists should be encouraged to participate and learn to be updated about emerging trends and advances in the field of pharmaceuticals and patient care. Pharmacies and pharmacists should affirm their responsibility and accountability as healthcare stakeholders in battling AMR in the community. They should create awareness campaigns to educate and create awareness among the public.

According to recent systematic reviews, the research on the enforcement of laws and their impact on reducing OTC access to antibiotics in LMICs is sparse [26]. In India, the state pharmacy councils may collaborate with the pharmacy institutions and encourage the students to research the dispensing practices not only limited to antibiotics but also relating to other drugs. They may volunteer the students to conduct surveys and awareness campaigns to educate the public.

Studies have shown that total antibiotic consumption in most countries has been linked to a higher incidence of AMR [25,26]. Anti-microbial stewardship interventions are effective in improving the appropriateness of antibiotic use and tailored stewardship programs are required to better control anti-microbial use and combat AMR [27]. As the ongoing persistence of AMR diminishes the efficacy of antibiotics, doctors ought to consider resorting to medications from the last line of defense, such as carbapenems and polymyxins. These medications may not always be easily accessible in developing nations due to high costs and are associated with different side effects [6]. Our study suggests that interventions should be taken (such as prescription retentions, regulatory inspections, media campaigns, and pharmacist education) to enforce stringent laws to prohibit unethical dispensing.

Limitations

As our study was conducted using the pharmacy questionnaire method with open-ended questions, the responses might be influenced by the Hawthorne effect (changes in the behavior/response of the studied pharmacists because they feel observed). Hence, a simulated patient method (an individual visiting a pharmacy simulating specific symptoms and requiring an antibiotic) in the population of interest is recommended for a yet more clear understanding of the antibiotic-dispensing patterns. However, both methods can provide complementary information [11].

## Conclusions

The results of our study have unearthed the concerning trend of inappropriate antibiotic-dispensing patterns in pharmacies in the Indian state of Andhra Pradesh. This disturbing trend is a primary contributing factor that could potentially exacerbate AMR in the community. Consequently, we advocate for an imperative implementation of rigorous regulatory measures and routine oversight to manage antibiotic dispensing, thereby, curbing unethical practices.
